# Photochemical Synthesis of Nucleoside Analogues from Cyclobutanones: Bicyclic and Isonucleosides

**DOI:** 10.3390/molecules15063816

**Published:** 2010-05-26

**Authors:** Mileina Jaffer, Abdelaziz Ebead, Edward Lee-Ruff

**Affiliations:** Department of Chemistry, York University, 4700 Keele Street, Toronto, Ontario, M2M 4J4, Canada

**Keywords:** cyclobutanone, oxacarbene, bicyclic nucleosides, isonucleosides, acyclic nucleoside

## Abstract

The preparation of two nucleoside analogues are reported. Both syntheses involve a key photochemical ring-expansion of cyclobutanones to an oxacarbene and its subsequent scavenging by 6-chloropurine. The synthesis of a bicyclic (locked) purine starts from a oxabicycloheptanone with a hydroxymethyl pendant. The preparation of an isonucleoside uses a cyclobutanone with an α-substituted 6-chloropurine. Irradiation of the latter produces an isonucleoside and acyclic nucleoside analogues.

## 1. Introduction

**Scheme 1:**
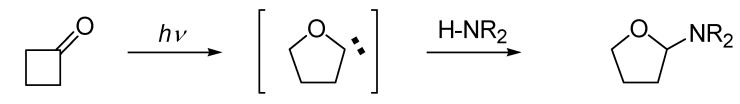
*N*-Glycosylation by Photochemical Ring-expansion of Cyclobutanones.

Structurally modified nucleosides represent an important class of medicinal compounds which have been found to behave as therapeutic agents and are currently used in pharmaceuticals as antitumour, antiviral, and antibiotic agents [1,2,3,4]. Structural modifications include the ribose, as well as the base moieties. The general synthetic protocols involve the use of monosaccharide chirons which are coupled to heterocycles employing a key glycosylation step which is often not stereoselective. During the last decade our group has developed a photochemical glycosylation method based on the photoisomerization of cyclobutanones to transient 2-tetrahydrofuranylidenes and their insertion into alcohols [5] or weakly acidic N-H functions (Scheme 1) [6,7,8,9,10,11,13]. 

The *N*-glycosylation procedure was exploited for the preparation of modified ribonucleosides [6,7,8,9,10,11,13]. Although anomeric mixtures were obtained, some stereoselectivity was observed, depending on the nature of the ring substituents. The advantages of this method are the mild conditions for coupling (neutral solutions), the retention of stereochemistry of the cyclobutanone ring substituents in the photoproducts, and the availability of substituted cyclobutanone precursors with defined stereochemistry using relatively simple methods [14,15]. These precursors can also be prepared in chiral modifications for their use in the preparation of chiral nucleosides.

In the current investigation we report the synthesis of two new nucleoside analogues, a bicyclic (“locked”) derivative and an isonucleoside, from two different cyclobutanone precursors. Furthermore, photocycloelimination of cyclobutanones results in the stereospecific formation of alkenes as a side reaction. The extent to which cycloelimination occurs is dependent on the electronic nature of the α-substituent in the ketone as well as solvent effects. Electron-withdrawing groups promote cyloelimination over photoisomerization [16]. We also report the stereoselective formation of an acyclic nucleoside analogue from the photocycloelimination of an α-nucleobase-substituted cyclobutanone.

## 2. Results and Discussion

### 2.1. Photochemical Synthesis of a Bicyclic Nucleoside Analogue

**Figure 1:**
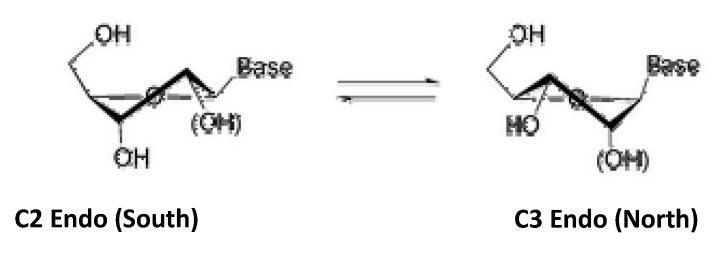
Nucleoside Conformations.

A class of structurally modified nucleosides consists of bicyclic or “locked” derivatives. In this class of nucleosides, there is an additional ring fused to the sugar moiety [17]. The five membered sugar rings found in nucleosides exist in a puckered geometry. Figure 1 depicts this geometry in either the “south” or the “north” conformation. The two conformers are in equilibrium. 

**Figure 2:**
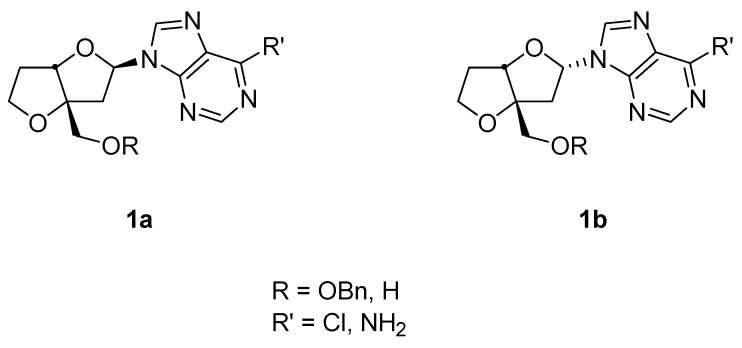
Target Bicyclic Derivatives.

The specific conformation is crucial for complementary binding with target molecules. An attractive feature of bicyclic derivatives is their restricted conformational flexibility [17]. Thus, the nucleoside may be locked in the active conformation (either north or south) required to show activity. Due to their decreased conformational flexibility, oligonucleotides incorporating these rigid nucleoside analogues have been observed to display increasing recognition of DNA sequences [17]. The ability of nucleotides containing locked nucleoside to be able to recognize DNA sequences have rendered these promising as drug candidates for antisense therapy. We have previously reported the photochemical ring-expansion of a series of bicyclic cyclobutanones and their carbene insertion reactions to 6-chloro-purine in order to explore this approach for the preparation bicyclic nucleoside analogues [13]. The earlier report was to demonstrate the proof of concept, but did not incorporate important structural features associated with substituents necessary for biological functions (i.e. hydroxymethyl, hydroxyl groups). In the current study we report the preparation of nucleoside analogues **1a **and **1b** (Figure 2) with the appropriate hydroxymethyl substituent for binding to the phosphorylating kinase receptor [18] using the photochemical protocol developed by our group.

**Scheme 2:**
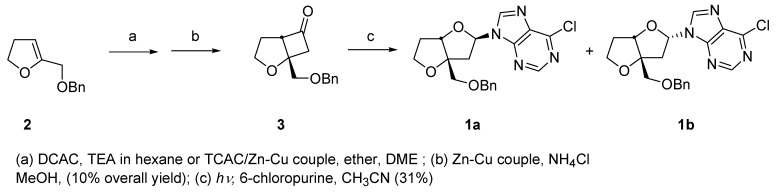
Preparation of a Bicyclic Nucleoside Analogue.

The bicycloheptanone **3** was prepared in two steps from the previously reported 2-benzyloxymethyl-4,5-dihydrofuran (**2**) [19] using the standard [2+2] dichloroketene cycloaddition and dechlorination sequence. Although the overall yield for the two steps is low (~10%) no optimization was carried out and significant amounts of starting **2** accompanied product formation. The regioselectivity for the cycloaddition follows previously observed ketene cycloadditions to vinyl ethers [20]. Irradiation of ketone **3** with 6-chloropurine in acetonitrile solution produced a diastereomeric mixture of two anomers **1a** and **1b** in a 2:1 ratio (30%) (Scheme 2). 

The remaining products consisted of polar materials which were not identified. Photocyclo-elimination is known to accompany ring-expansion, the latter pathway leading to a substituted ketene which could oligomerize or produce carboxylic acids on work up [16]. The regioselectivity for insertion of the oxacarbene to the N-9 position has been established from previous studies [6,8,9,10,11] with the predominant presence of the N-9(H) tautomer in solution. The major epimer was shown to possess the *exo* stereochemistry on the basis of 1D and 2D (HSQC, COSY and NOESY). For **1a **the NOE correlation was established between the anomeric and the bridgehead protons. For **1b** there was no observable correlation between these respective protons. The *cis*-fused assignment of both epimers was established by NOE correlation between the bridgehead and methylene protons. This observation is consistent with the retention of the ring substituent stereochemistry in these photochemical ring-expansions of cyclobutanones.

The preference for formation of the *exo*-adduct is likely due to the less sterically encumbered *exo*-approach of the carbene scavenger. The conversion of the chloro-nucleosides to the adenine derivatives, **1a** and **1b **(R’ = NH_2_) as well as deprotection of the alcohol group are straightforward steps. Our investigations are continuing on extensions to the homologous dihydropyran series, although these would be more conformationally flexible than the corresponding furan series.

### 2.2. Synthesis of Acyclic and Isonucleoside Analogues from the same Cyclobutanone Precursor

Isonucleosides are analogues in which the substituents of the ribose moiety are transposed to different positions in the sugar ring from their natural counterpart [21]. Acyclic nucleosides incorporate a non cyclic ribose equivalent [22]. Many of these exhibit antiviral activity such as acyclovir which is used in the treatment of most forms of herpes.

**Scheme 3:**
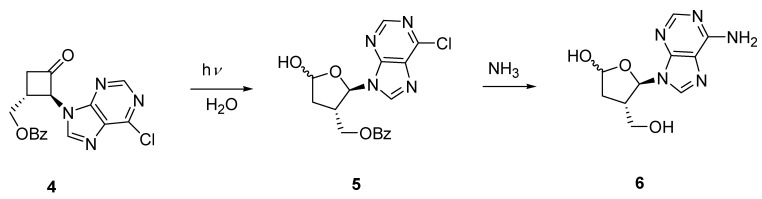
Preparation of an Isonucleoside.

In a separate study involving the preparation of cyclobutane carbocyclic analogues of nucleosides we synthesized ketone **4** by way of *N*-alkylation of 2-bromo-3-benzoylxycyclobutanone [10] with 6-chloropurine [23]. The stereochemistry of **4** was confirmed from the X-ray crystal structure analysis of one of its derivatives. Photochemical ring-expansion in the presence of water should result in hemiacetal **5** which on deprotection and aminolysis, carried out in a one pot reaction, would give isoadenosine **6 **(Scheme 3). Regioselectivity would be expected from scission of the more substituted carbonyl carbon bond.

**Scheme 4:**
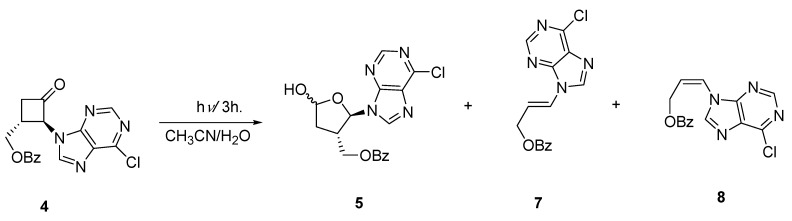
Photochemical Production of an Isonucleoside and Acyclic Derivatives.

Irradiation of **4** in acetonitrile containing 10 stoichiometric equivalents of water produced, in addition to **5 **(20%), geometric isomers **7** (11%) and **8** (64%) derived from cycloelimination of ketene Scheme 4). The hemiacetal **5 **was formed as a single stereoisomer as was evident by the simple doublet signal observed for the anomeric proton of the aminoacetal group, although the relative stereochemistry of the hemiacetal group could not be unambiguously assigned. The formation of the *cis*-isomer **8 **as the major product was unexpected as the fragmentation of ketene from electronically excited cyclobutanones is known to be a concerted process originating from the singlet state and leads to alkenes with retention of stereochemistry. 

Photocycloelimination of ketene is regioselective, involving cleavage of the more substituted α-carbon-carbonyl carbon bond similar to the regioselectivity of the ring-expansion reaction. The extent to which each of these competitive processes occur is influenced by the nature of the α-substituent with electron-withdrawing groups favouring cycloeliminations and electron-donating groups giving ring-expansion oxacarbene photoadducts [16]. The distribution of these two competitive pathways is also affected by solvent polarity, with polar solvents favouring ring-expansion and non-polar solvents giving rise to cycloeliminations [16]. The solvent effects on the distribution of products is attributed to reversible formation of the more polar oxacarbene intermediate [16].

**Scheme 5:**
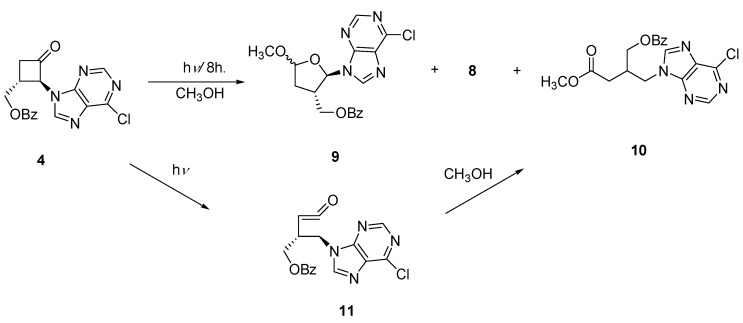
Photochemical Production of an Isonucleoside and Acyclic Derivative.

In order to maximize the yields of the ring-expansion photoadducts, the photolysis of **4** was carried out in more polar methanol solutions (Scheme 5). The yield for the ring-expansion photoadduct **9** increased to 40%. Under these conditions a small amount of **8** (13%) was observed with no detectable presence of **7**. However, a new photoadduct **10** was observed in 21% yield. 

The latter is likely formed from a Norrish Type I reaction involving a transient ketene **11**. This mode for deactivation is unusual for cyclobutanones which yield photoproducts originating from the singlet state on direct irradiation. Ketene formation from Norrish I reactions normally originate from the triplet state in acyclic and medium size cycloalkanones [24]. Diester **10** would serve as an important precursor to potential biologically active acyclic nucleosides on reduction-deprotection and aminolysis.

The purine substituted alkenes **7** and **8 **are also important precursors to other acyclic nucleoside as well as cyclopropane analogues. The effect of less polar solvents such as benzene and acetone on the photochemistry of ketone **4** was investigated with the objective of maximizing the cycloelimination pathway. Both solvents yielded alkenes **7** and **8** as the only identifiable products, however the total yield for **7** and **8** was lower than that obtained in acetonitrile. In benzene *trans*-**7** exceeded *cis*-**8** by a factor of 2:1. For acetone, a known triplet sensitizer, the distribution of **7** and **8** was about the same. The unusual non-steroselective photocycloelimination observed in the case of ketone **4** is likely the result of concurrent photoisomerization between **7** and **8** as was shown by independent experiments under the conditions of direct or sensitized irradiation.

## 3. Experimental

### 3.1. General

Photolyses were carried out using a Hanovia 450-W medium pressure mercury arc lamp in a water-cooled quartz immersion well. Pyrex tubes containing the samples were strapped around this well, and the assembly was immersed in an ice-water bath. The samples were purged with argon for 30 min prior to irradiation. All solvents used were dried and distilled. Proton NMR spectra were obtained on a Bruker ARX-400 spectrometer and ^13^C NMR was carried out on a Bruker 300 spectrometer. Mass Spectra were collected (EI mode) using a Varian Series 4000 GC/MS/MS spectrometer. 

### 3.2. 1-(Benzyloxymethyl)-7,7-dichloro-2-oxabicyclo[3.2.0]heptan-6-one

#### 3.2.1. Method A

Enol ether **2** [20] (2.36 g, 0.0124 mol) was dissolved in hexanes (60 mL) and stirred at room temperature. Dichloroacetyl chloride (1.92 g, 0.015 mol) was added and the mixture was allowed to reflux for 20 min. Triethylamine (1.50 g, 0.015 mol) in hexanes (30 mL) was then added dropwise to the mixture. The reaction was allowed to stir for 1 h and quenched with cold H_2_O (30 mL). The organic layer was washed with saturated NaHCO_3_, dried over Na_2_SO_4_, and the solvent was removed under vacuum to give the title compound in 21% yield. IR (1,805 cm^-1^) confirmed the presence of the cyclobutanone carbonyl group. The crude material was used immediately for the dechlorination step without purification.

#### 3.2.2. Method B

To a solution of alkene **2** (4.022 g, 0.0212 mol) in ether (80 mL) and DME (11 mL) was added Zn/Cu couple [25] (4.29 g). The mixture was heated to reflux and trichloroacetyl chloride (TCAC) (10.409 g, 0.05724 mol) was added dropwise to the mixture. The mixture was allowed to reflux overnight. Zinc precipitate was filtered and the ether was evaporated under reduced pressure. The residue was dissolved in hexanes and the organic layer was washed twice with saturated NaHCO_3_ solution. The organic layer was then washed with brine, dried over Na_2_SO_4_, and the solvent was removed under reduced pressure to give the title compound which showed identical spectral properties as the sample obtained from method A. 

### 3.3. 1-(Benzyloxymethyl)- 2-oxabicyclo[3.2.0]heptan-6-one (3)

#### 3.3.1. Method A

Dichloroketone (0.372 g, 0.00123 mol) obtained above was stirred in MeOH (30 mL) at 0 ºC. To this solution, zinc powder (0.88 g, 0.013 mol) and ammonium chloride (0.48 g, 0.009 mol) was added. The mixture was allowed to stir and warm to room temperature overnight. The zinc was filtered and the solvent was removed under reduced pressure. The residue was dissolved in ether (3 × 20 mL) and the organic layer was washed with 2 × 15 mL saturated NaHCO_3_, dried over Na_2_SO_4_, and the solvent removed under reduced pressure to give an oil which was purified by preparative TLC (30% ethyl acetate in hexanes). Yield 10%. IR (cm^-1^): 1807; ^1^H-NMR (CDCl_3_): δ 7.30–7.38 (m, 5H), 4.66–4.70 (m, 2H), 4.21–4.28 (m, 1H), 3.97-4.03 (m, 1H), 3.82-3.84 (m, 2H), 3.64-3.68 (m, 1H), 3.21-3.29 (dd, *J *= 5.4 Hz, 18 Hz, 1H), 2.93-3.00 (dd, *J* = 2.1 Hz, 18 Hz, 1H), 1.90–2.22 (m, 2H); ^13^C-NMR (300 MHz, CDCl_3_): δ 214 (C=O, C-1), 65.38 (CH, C-2), 29.4 (CH_2_, C-3), 69.2 (CH_2_, C-4), 29.9 (C, C-5), 53.2 (CH_2_, C-6), 71.7 (CH_2_, C-7), 73.6 (CH_2_, C-8) 138 (C, C-9), 127.8-130.6 (CH, C-10); ^1^H- and ^13^C-NMR assignments from COSY, HSQC and NOESY data are given in Table 1; MS (EI) *m/z* 232 (M^+^). 

**Table 1:** Assignment of ^13^C and ^1^H Chemical Shift Values from COSY, HSQC and NOESY Data.

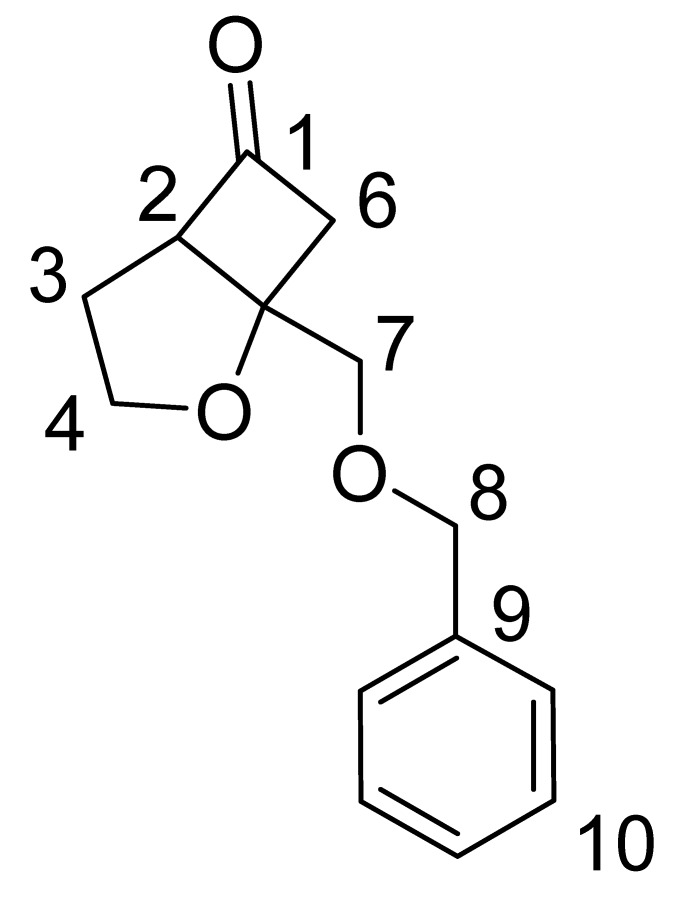		
Carbon No.	^13^C δ (ppm)	^1^H δ (ppm)
1	214	
2	65.4	3.64–3.68 (m, 1H)
3	29.37	1.90–2.22 (m, 2H)
4	69.20	3.97–4.03 (m, 1H) 4.21-4.28 (m, 1H)
5	29.9	
6	53.1	3.21–3.29 (dd, *J* = 5.4 Hz, 18Hz, 1H) 2.93–3.00 (dd, *J *= 2.1 Hz, 18 Hz, 1H)
7	71.6	3.82–3.89 (m, 2H)
8	73.5	4.66–4.70 (m, 2H)
9	138	
10	127.7–130.6	7.30–7.38 (m, 5H)

#### 3.3.2. Method B

To a stirring mixture of 1-(benzyloxymethyl)-7,7-dichloro-2-oxabicyclo[3.2.0]heptan-6-one obtained above (1.75 g, 0.0096 mol) and ammonium chloride (2.59 g, 0.048 mol) in methanol (41 mL) was added zinc-copper couple (1.90 g) at room temperature. The reaction mixture was stirred for 1 h. The zinc precipitate was filtered and the filtrate was concentrated. The residue was dissolved in DCM, and the solution was washed with brine, dried over anhydrous sodium sulphate, filtered, and concentrated. The residue was purified by preparative TLC (30% ethyl acetate-hexanes). Yield 10%. The sample obtained showed identical spectral properties with those of the sample obtained from method A. 

### 3.4. 9-(3a-(Benzyloxymethyl)dihydrofuro[3,2-b]furan-2-yl)-6-chloro-9H-purine (1a and 1b)

A solution of ketone **3** (0.085 g, 0.00037 mol) and 6-chloropurine (0.085 g, 0.0006 mol) in acetonitrile (80 mL) was irradiated for 6 h in a pyrex tube. The solvent was removed under reduced pressure and the residue dissolved in chloroform. The photolysate were purified by preparative TLC (70% ethyl acetate-hexanes) giving **1a** (20%) and **1b** (10%) as oils. 

*Nucleoside* **1a**: ^1^H-NMR (CDCl_3_, 400 MHz): δ 8.70 (s, 1H), 8.45 (s, 1H), 7.25–7.37 (m, 5H), 6.53–6.55 (dd, *J* = 5.2 Hz, 7.2 Hz, 1H), 4.90 (d, *J* = 4.4 Hz, 1H), 4.5 (*AB *q, *J* = 12 Hz, 2H), 4.04–4.14 (m, 2H), 3.66 (d, *J* = 9.6 Hz, 1H), 3.53 (d, *J* = 9.6 Hz, 1H), 3.02 (dd, *J* =5.2 Hz, 14.4 Hz, 1H), 2.81 (dd, *J *= 7.2 Hz, 14.4 Hz, 1H), 2.12–2.23 (m, 2H);^ 13^C-NMR (CDCl_3_, 300 MHz): δ 92.2 (CH, C-1), 33.2 (CH_2_, C-2), 73.6 (CH_2_, C-3), 70.7 (CH_2_, C-5), 67.9 (CH_2_, C-4), 76.6 (C, C-7), 41.914 (CH_2_, C-8), 86.1 (CH, C-9), 126.0-127.8 (CH, C-6), 143.5 (CH, C-10), 151.9 (CH, C-11); MS (EI) *m/z* 388 (M^+^, ^37^Cl), 386 (M^+^, ^35^Cl); ^1^H- and ^13^C-NMR assignments from COSY, HSQC and NOESY data are given in Table 2.

*Nucleoside* **1b**: ^1^H-NMR (CDCl_3_, 300 MHz): δ 8.59 (s, 1H), 8.79 (s, 1H), 7.28–7.40 (m, 5H), 6.47–6.49 (dxd, *J* = 3.9 Hz, 7.8 Hz, 1H), 4.73 (d, *J* = 2.4 Hz, 1H), 4.63 (s, 2H), 4.09 (m, 2H), 3.63 (d, *J* = 9.6 Hz, 1H), 3.54 (d, *J* = 9.6 Hz, 1H), 2.99 (dd, *J* =7.8 Hz, 14.7 Hz, 1H), 2.76 (dd, *J* = 3.6 Hz, 14.7 Hz, 1H), 2.13-2.19 (m, 2H); ^13^C NMR (CDCl_3_, 300 MHz): δ 87.266 (CH, C-1), 32.777 (CH_2_, C-2), 73.738 (CH_2_, C-3), 68.196 (CH_2_, C-4), 70.931 (CH_2_, C-5), 76.00 (C, C-7), 40.108 (CH_2_, C-8), 84.240 (CH, C-9), 143.981 (CH, C-10), 127.304-128.577 (CH, C-6), 152.214 (CH, C-11); MS (EI) *m/z* 388 (M^+^, ^37^Cl), 386 (M^+^, ^35^Cl); ^1^H- and ^13^C-NMR assignments from COSY, HSQC and NOESY data are given in Table 2.

**Table 2:** Assignment of ^13^C and ^1^H Chemical Shift Values from COSY, HSQC and NOESY Data.

Major nucleoside 1a		
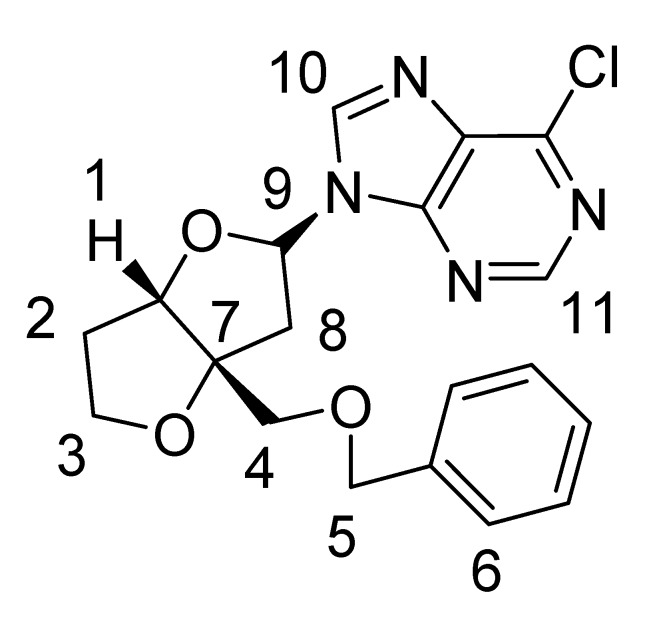		
Carbon No.	^13^C δ (ppm)	^1^H δ (ppm)
1	92.2	4.90 (d, *J *= 4.4 Hz, 1H)
2	33.2	2.12–2.23 (m, 2H)
3	73.6	4.04–4.14 (m, 2H)
4	67.9	3.66 (d, *J* = 9.6 Hz, 1H), 3.53 (d, *J* = 9.6 Hz, 1H)
5	70.7	4.5 (*AB *q, *J* = 12 Hz, 2H)
6	126.0-127.8	7.25-7.37 (m, 5H)
7	76.6	
8	41.9	2.81 (dd, *J* = 7.2 Hz, 14.4 Hz, 1H)3.02 (dd, *J* = 5.2 Hz, 14.8 Hz, 1H)
9	86.1	6.53-6.55 (dd, *J *= 5.2 Hz, 7.2 Hz, 1H)
10	143.5	8.45 (s, 1H)
11	151.9	8.70 (s, 1H)
**Minor nucleoside 1b:**		
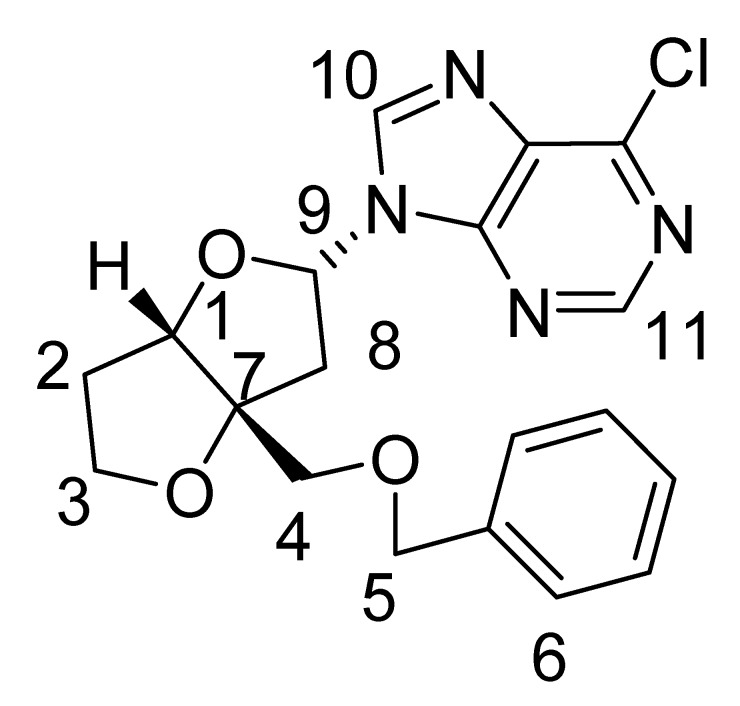		
**Carbon No.**	**^13^C δ (ppm)**	**^1^H δ (ppm)**
1	87.3	4.73 (d, *J *=2.4 Hz, 1H)
2	32.8	2.13–2.19 (m, 2H)
3	73.7	4.09 (m, 2H)
4	68.2	3.63 (d, *J* = 9.6 Hz, 1H), 3.54 (d, *J* = 9.6 Hz, 1H)
5	70.9	4.63 (s, 2H)
6	127.3–128.6	7.28–7.40 (m, 5H)
7	76.0	
8	40.1	2.99 (dd, *J *=7.8 Hz, 14.7 Hz, 1H)2.76 (dd, *J *= 3.6 Hz, 14.7 Hz, 1H)
9	84.2	6.47–6.49 (dxd, *J* = 3.9 Hz, 7.8 Hz, 1H)
10	144.0	8.59 (s, 1H)
11	152.2	8.79 (s, 1H)

### 3.5. Cyclobutanone ***4***

This ketone was prepared by a two step sequence involving α-bromination followed by *N*-alkylation with 6-chloropurine [23]. Regioisomers of the *N*-7 and *N*-9 (compound **9**) alkylated products were formed and their structures determined by X-ray single crystal diffraction [23]. m.p. 60–62 ºC; IR (cm^-1^) 1799 (C=O of cyclobutanone); 1715 (C=O of ester); ^1^H-NMR (CDCl_3_): δ 8.65 (s, 1H), 8.17 (s, 1H), 7.90–7.88 (d, *J *= 8.0 Hz, 2H), 7.58–7.55 (t, *J *= 7.0 Hz, 1H), 7.42–7.38 (t, *J *= 7.8 Hz, 2H), 5.78–5.76 (d, *J *= 7.6 Hz, 1H), 4.77–4.76 (d, *J *= 5.2 Hz, 2H), 3.60–3.54 (m, 1H), 3.47-3.40 (m, 1H), 3.26–3.19 (dd, *J *= 8.8 Hz, 17.6 Hz, 1H); ^13^C-NMR (CDCl_3_): δ 196.9, 166.6, 152.3, 151.6, 151.5, 144.0, 133.8, 131.6, 129.7, 129.2, 128.8, 68.9, 65.0, 45.3, 33.3.

### 3.6. Photolysis of Ketone ***4*** in Acetonitrile spiked with Water

A solution of ketone **4** (71 mg, 0.2 mmol) in 45 mL of acetonitrile and 0.036 mL (2.0 mmol) of water was irradiated for 3 h. The solvent was removed under vacuum and the residue purified by preparative TLC (60% ethyl acetate-hexane) to give 15 mg (20.2%) of diastereomeric mixture of hemiacetal **5**, 7 mg (11.1%) of *trans*-alkene **7** and 40 mg (63.5%) of the *cis*-alkene **8**. 

*Benzoic acid 2-(6-chloro-purin-9-yl)-5-hydroxy-tetrahydro-furan-3-ylmethyl ester*** (5)***.* ^1^H-NMR (CDCl_3_): δ 8.76 (s, 1H), 8.29 (s, 1H), 8.13–8.11 (d, *J *= 8.0 Hz, 2H), 7.64–7.59 (t, *J *= 6.4 Hz, 1H), 7.48–7.44 (t, *J *= 8.0 Hz, 3H), 6.56–6.55 (d, *J *= 4.0 Hz, 1H), 4.70–4.68 (dd, *J *= 4.0 Hz, 12.0 Hz, 1H), 4.55–4.50 (dd, *J *= 6.4 Hz, 11.6 Hz, 1H), 3.80–3.78 (m, 1H), 3.66–3.59 (dd, *J *= 10.0 Hz, 18.0 Hz, 1H), 2.80–2.74 (m, 1H); MS: *m/z* 361 (M^+^-OH, ^37^Cl), 386 (M^+^-OH, ^35^Cl).

E-1’-(6-Chloropurin-9-yl)-2’-benzoyloxyethene (**7**). ^1^H NMR (CDCl_3_): δ 8.82 (s, 1H), 8.32 (s, 1H), 8.10-8.08 (d, *J *= 8.0 Hz, 2H), 7.61-7.58 (t, *J *= 7.0 Hz, 1H), 7.49-7.45 (t, *J *= 7.6 Hz, 2H), 7.41–7.38 (d, *J *= 14.4 Hz, 1H), 6.94-6.88 (ddd, *J *= 6.4, 14.4, 20.8 Hz, 1H), 5.08-5.07 (d, *J *= 6.0 Hz, 2H); ^13^C NMR (CDCl_3_): δ 166.3, 152.8, 151.8, 151.0, 143.0, 133.5, 132.4, 130.3, 129.9, 128.7, 124.0, 117.1, 62.7; MS: *m/z* 316 (M^+^, ^37^Cl), 314 (M^+^, ^35^Cl).

Z-1’-(6-Chloropurin-9-yl)-2’-benzoyloxyethene (**8**).^ 1^H-NMR (CDCl_3_): δ 8.80 (s, 1H), 8.41 (s, 1H), 7.97-7.96 (d, *J *= 7.8 Hz, 2H), 7.59-7.56 (t, *J *= 7.5 Hz, 1H), 7.45-7.42 (t, *J *= 7.8 Hz, 2H), 7.11-7.10 (d, *J *= 9.0 Hz, 1H), 6.16-6.12 (q, *J *= 7.6, 15.6 Hz, 1H), 5.03-5.02 (d, *J *= 6.6 Hz, 2H); ^13^C-NMR (CDCl_3_): δ 166.2, 152.9, 151.9, 151.8, 144.4, 133.7, 131.4, 129.8, 129.4, 128.7, 123.0, 122.1, 59.8; MS: *m/z* 316 (M^+^, ^37^Cl), 314 (M^+^, ^35^Cl).

### 3.7. Photolysis of Ketone ***4*** in Methanol

A solution of ketone **4** (60 mg, 0.17 mmol) in methanol (50 mL) was irradiated for 8 h. Evaporation of the solvent under vacuum followed by chromatographic purification (gradient solvent mixture from 40% ethyl acetate-hexane to 60% ethyl acetate-hexane) yielded 7 mg (13.2%) of *cis*-alkene **8**, 14 mg (21.2%) of diester **10**, and 26 mg (39.3%) methoxyacetal **9** as a diastereomeric mixture which was further separated by preparative TLC (40% ethyl acetate-hexane).

*Benzoic acid 2-(6-chloro-purin-9-yl)-5-methoxy-tetrahydro-furan-3-ylmethyl ester*** (9) (less polar anomer).** ^1^H-NMR (CDCl_3_) :δ 8.59 (s, 1H), 8.47 (s, 1H), 7.64-7.62 (d, *J *= 7.6 Hz, 2H), 7.53–7.49 (t, *J *= 7.4 Hz, 1H), 7.31–7.27 (t, *J *= 7.2 Hz, 2H), 6.57–6.55 (d, *J *= 5.6 Hz, 1H), 5.30-5.27 (m, 1H), 4.67–4.57 (dd, *J *= 4.6, 11.0 Hz, 1H), 4.47–4.42 (m, 1H), 3.48-3.42 (m with overlapping s, 4H), 2.47-2.44 (dd, *J *= 7.6, 12.8 Hz, 1H), 2.20-2.13 (m, 1H); MS: *m/z* 390 (M^+^, ^37^Cl), 388 (M^+^, ^35^Cl).

*Benzoic acid 2-(6-chloro-purin-9-yl)-5-methoxy-tetrahydro-furan-3-ylmethyl ester*** (9) (more polar anomer).** ^1^H- NMR (CDCl_3_): δ 8.59 (s, 1H), 8.26 (s, 1H), 7.84–7.82 (d, *J *= 7.6 Hz, 2H), 7.50–7.45 (t, *J *= 7.8 Hz, 1H), 7.40–7.35 (t, *J *= 7.8 Hz, 2H), 6.40–6.39 (d, *J *= 4.0 Hz, 1H), 5.44–5.43 (d, *J *= 4.8 Hz, 1H), 4.59–4.57 (d, *J *= 7.6 Hz, 2H), 3.48-3.42 (m with overlapping s, 4H), 2.80–2.73 (m, 1H), 2.03–1.99 (dd, *J *= 3.6, 14.0 Hz, 1H); MS: *m/z* 390 (M^+^, ^37^Cl), 388 (M^+^, ^35^Cl).

*Benzoic acid 2-(6-chloro-purin-9-ylmethyl)-3-methoxycarbonyl-propyl ester*** (10)**. ^1^H-NMR (CDCl_3_): δ 8.70 (s, 1H), 8.23 (s, 1H), 7.91–7.90 (d, *J *= 7.6 Hz, 2H), 7.60–7.57 (t, *J *= 7.0 Hz, 1H), 7.46–7.43 (t, *J *= 7.8 Hz, 2H), 4.58–4.47 (m, 2H), 4.41-4.31 (m, 2H), 3.12–3.03 (m, 1H), 2.59–2.47 (m, 2H); ^13^C-NMR (CDCl_3_): δ 171.7, 166.2, 152.3, 151.4, 145.7, 133.7, 131.6, 130.2, 129.6, 129.4, 128.7, 64.7, 52.3, 45.4, 35.9, 33.7; MS: *m/z* 390 (M^+^, ^37^Cl), 388 (M^+^, ^35^Cl).

### 3.8. Photolysis of Ketone ***4*** in Acetone

A solution containing 100 mg (0.28 mmol) of ketone **4** in 90 mL of acetone was irradiated for 6 h. The solvent was removed and the residue purified by preparative TLC (30% ethyl acetate-hexane) to give 18 mg (20.4%) of *trans*-alkene **7**, and 21 mg (23.9%) of *cis*-alkene **8**. The spectral properties for each of these fractions were identical for the corresponding derivatives reported above. 

### 3.9. Photolysis of Ketone ***4*** in Benzene

A solution of ketone **4** (120 mg, 0.34 mmol) in 80 mL of benzene was irradiated for 3 h. Removing of the solvent under vacuum followed by chromatographic purification (30% ethyl acetate-hexane) yielded 20 mg (18.7%) of *cis*-alkene **8** and 35 mg (32.7%) of *trans*-alkene **7** with identical spectral properties as reported above.

## 4. Conclusions

The direct irradiation of the bicyclic cyclobutanone **3** proceeds by isomerisation to a transient oxacarbene which can insert into the *N*-9 N-H bond of 6-chloropurine to give the corresponding bicyclic nucleoside analogues. The choice of 6-chloropurine was based on its solubility in common organic solvents such as acetonitrile or THF, ensuring homogeneity in order to maximize photochemical efficiency. Furthermore, nucleophilic aryl substitution of the C(6)-Cl function by NH_2_ has been shown to occur under mild conditions in order to access the adenine derivatives [6]. While yields are quite modest, this approach represents one of the most simple routes to these derivatives. Another cyclobutanone **4** in which the purine nucleobase is attached undergoes photochemical ring-expansion with water or methanol to give photoadducts which represent the isonucleoside class of derivatives. A competing side reaction involving cycloelimination yields purinyl base substituted alkenes, the distribution of which is solvent dependent. These compounds are of interest as intermediates in the synthesis of cyclopropane carbocyclic nucleosides. The photolysis of **4** in methanol produced an unexpected Norrish I photoadduct **10** which on hydride reduction would generate the corresponding diol representing an acyclic nucleoside analogue. These latter studies are currently being extended to include pyrimidine nucleobases as part of a general program for the preparation of cyclobutane based nucleoside analogues.
